# Genetic Diversity of Human Enterovirus 68 Strains Isolated in Kenya Using the Hypervariable 3′- End of VP1 Gene

**DOI:** 10.1371/journal.pone.0102866

**Published:** 2014-07-23

**Authors:** Silvanos M. Opanda, Fred Wamunyokoli, Samoel Khamadi, Rodney Coldren, Wallace D. Bulimo

**Affiliations:** 1 Department of Emerging Infectious Diseases (DEID), United States Army Medical Research Unit-Kenya (USAMRU-K), Nairobi, Kenya; 2 The Kenya Medical Research Institute (KEMRI), Nairobi, Kenya; 3 Department of Biochemistry, Jomo Kenyatta University of Agriculture and Technology, (JKUAT), Nairobi, Kenya; 4 College of Health Sciences (COHES), Jomo Kenyatta University of Agriculture and Technology, (JKUAT), Nairobi, Kenya; University of Illinois at Chicago, United States of America

## Abstract

Reports of increasing worldwide circulation of human enterovirus-68 (EV68) are well documented. Despite health concerns posed by resurgence of these viruses, little is known about EV68 strains circulating in Kenya. In this study, we characterized 13 EV68 strains isolated in Kenya between 2008 and 2011 based on the Hypervariable 3′- end of the VP1 gene. Viral RNA was extracted from the isolates and partial VP1 gene amplified by RT-PCR, followed by nucleotide sequencing. Alignment of deduced amino acid sequences revealed substitutions in Kenyan EV68 isolates absent in the prototype reference strain (Fermon). The majority of these changes were present in the BC and DE-loop regions, which are associated with viral antigenicity and virulence. The Kenyan strains exhibited high sequence homology with respect to those from other countries. Natural selection analysis based on the VP1 region showed that the Kenyan EV68 isolates were under purifying selection. Phylogenetic analysis revealed that majority (84.6%) of the Kenyan strains belonged to clade A, while a minority belonged to clades B and C. Overall, our results illustrate that although EV68 strains isolated in Kenya were genetically and antigenically divergent from the prototype strain (Fermon), they were closely related to those circulating in other countries, suggesting worldwide transmissibility. Further, the presence of shared mutations by Kenyan EV68 strains and those isolated in other countries, indicates evolution in the VP1 region may be contributing to increased worldwide detection of the viruses. This is the first study to document circulation of EV68 in Kenya.

## Introduction

Human enterovirus-68 (Enterovirus-68, also EV68) was originally isolated in the USA from patients with acute respiratory illnesses [1]–[3]. It is classified in the human enterovirus D species, along with enterovirus-70, enterovirus-94 and enterovirus-111 serotypes [4], [5]. Unlike other enteroviruses, EV68 shares common properties with rhinoviruses [2], [6]. These properties include acid lability, growth at low optimal temperatures (∼33°C) and isolation almost solely from respiratory samples [1]–[3], [6], [7]. The full spectrum of clinical diseases caused by EV68 is still unclear. However, the virus has been associated with acute respiratory tract illnesses including pneumonia and bronchiolitis [4], [8]–[11]. Other studies have also linked the virus with infection of the central nervous system [3], [12].

Since its first isolation, EV68 has been detected only sporadically [3]. However, in recent times, increased reports of worldwide circulation of these viruses have been published. Outbreaks of EV68 infections have been reported in France (2008), Italy (2008–2009), Philippines (2008–2009), Japan (2010), the United States of America (2009, 2010), Thailand (2006–2011), Netherlands (2008–2010), the United Kingdom (2009–2010), New Zealand (2010), South Africa (2001), Gambia (2008) and Senegal (2010), among other countries [4], [13]–[15]. In the bulk of these reports, children were most vulnerable [3]. However, recent studies have also shown EV68 infection in adults [10].

Enterovirus-68 genome consists of a positive single-stranded RNA molecule of about 7.5 kb encased in an icosahedral capsid. As with other picornaviruses, it is composed of a single open reading frame (ORF) flanked by un-translated regions (UTR) at the 3′ and 5′ ends [16]. The ORF encodes a precursor polyprotein that is cleaved by viral proteases to yield structural proteins VP1–VP4 and non-structural proteins; 2A, 2B, 2C, 3A, 3B, 3C and 3D [16], [17]. The VP1, VP2, and VP3 structural proteins are highly variable and form major epitopes of the virus exposed to the host immune system and neutralizing antibodies [16]. The VP1 contains serotype specific neutralization site, the BC-loop, a region found at the carboxyl end of the protein and associated with viral antigenicity [18] hence it is a key genotype determinant. Genotyping coupled with phylogenetic analysis based on sequence data of the VP1 region, has been shown to discriminate lineages within serotypes and detect new or emerging strains [19]. Consequently, among the structural proteins the VP1 has been used extensively in molecular analysis of enteroviruses for epidemiological and surveillance purposes.

The molecular mechanism underlying sudden upsurge of EV68 infections in many parts of the world remains obscure [20]. Understanding evolutionary aspects of EV68 strains isolated in different countries may be important in revealing biological factors contributing to resurgence of this virus. In this study, we present the first genetic analysis of EV68 viruses found in Kenya.

## Materials and Methods

### Study sites, inclusion criteria, clinical parameters and virus isolation

Human enterovirus (HEV) isolates used in this study were retrieved from archives of the respiratory virus surveillance program in the Department of Emerging Infectious Diseases (DEID) of the United States Army Medical Research Unit-Kenya (USAMRU-K), at the National Influenza Center, within the Kenya Medical Research Institute (KEMRI). The program surveillance network was selected to include disparate geographic regions and population demographics across the country and comprised Mbagathi, New Nyanza, Malindi, Isiolo, Mombasa, Port Reitz, Alupe, Kisii and Kericho District Hospitals.

Inclusion criteria included being an outpatient, >2 months in age and having influenza-like-illness (ILI) symptoms. Demographic information including age, sex, occupation, workplace and residence history were ascertained for all participants. Clinical parameters and symptoms including recent history of ILI, cough, difficulty in breathing, chills, sore throat, muscle aches, retro-orbital pain, malaise, vomiting, neurological symptoms, abdominal pain, nasal stuffiness, runny nose, sputum production, headache, joint pain, fatigue, diarrhea and bleeding were documented.

Nasopharyngeal specimens were collected and viruses isolated using RD cells (ATCC® CCL-136) prepared in culture tubes (Nunc, Denmark). Each tube containing the cell line in Dulbecco’s minimum essential medium (Life Technologies, USA) was inoculated with 100 µl of clinical specimen and incubated at 33°C under 5% CO2, and virus growth monitored with reference to cytopathic effects (CPEs) for up to 2 weeks. The presence of HEV in culture was confirmed by indirect fluorescent antibody test using Millipore Light Diagnostics™ Kit (Millipore Corporation, USA) according to the manufacturer’s instructions.

### Ethics Statement

Before obtaining specimens from patients, the study objectives were verbally explained to the patients or their parents/guardians if they were critically ill or were children. After verbal explanation by study personnel and having been given time to read and understand the questionnaire in a language they understood, all participants were required to sign a duplicate written informed consent form allowing for collection and testing of a nasopharyngeal specimen before specimens were obtained. Those who could not read or write were read for by an independent witness in a language they understood and the witness was able to guide them through the consent process and in appending their signatures/thumb print on the consent form. Patients were allowed to withdraw consent at any point during the study. Two pairs of consent forms were signed for each patient. The first copy was retained by the patient/guardian and the other copy was kept on record for regulatory review. For children who could not provide consent because of their age, both verbal and written consent were sought from their parents or legal guardians. Two ethical review boards, viz: (1) the Walter Reed Army Institute of Research (WRAIR) Institutional Review Board (IRB) and (2) the Kenya Medical Research Institute (KEMRI) Ethics Review Committee (ERC) specifically approved this study and consent procedure under protocol approvals WRAIR#1991 and KEMRI SSC#2383 respectively.

### RNA Extraction, RT-PCR and Sequencing

RNA was extracted from 100 µL of infected RD culture supernatant with a QIAmp Viral RNA Mini Kit (Qiagen, Inc., USA) according to the manufacturer’s specifications. Partial VP1 gene was amplified by RT-PCR as previously described [15], [21], [22]. The PCR products were electrophoresed on a 1% Agarose gel (Sigma-Aldrich Co., USA), stained with ethidium bromide (0.5 µg/ml) (Sigma-Aldrich Co., USA) and DNA amplicons visualized using the Alpha Imager (Alpha Innotech, USA) in accordance with the manufacturer’s instructions. The amplicons were purified using Exonuclease I/Shrimp Alkaline Phosphatase (ExoSap-IT) enzyme (Affymetrix, USA) and sequenced directly on both strands with the same primers used in the PCR, on an automated ABI 3500×L Genetic Analyzer (Applied Biosystems, USA). Cycle sequencing was performed using the Big Dye Terminator v3.1 sequencing kit (Applied Biosystems, USA), which incorporates fluorescent-labeled dideoxy-chain terminators and normal deoxynucleotides.

### Sequence Analysis

VP1 DNA nucleotide sequence fragments were edited and assembled into consensus contigs using DNA baser version 3.2 [23]. Serotype identity of the isolates was determined by pair-wise comparisons, using CLC genomics Workbench v6.5 software (CLC bio, Denmark). An isolate was determined to be EV68 if it shared ≥75% nucleotide identity (85% amino acid similarity) with the prototype strain (Fermon) [17], [24]. EV68 VP1 sequences were compared to those previously published and deposited in GenBank. Multiple sequence alignment was performed using Muscle v3.8 software [25]. The mean d_N_/d_S_ (ω) value and selection pressure at individual codon sites were estimated by the single likelihood ancestor counting (SLAC) and fixed effects likelihood (FEL) methods [26] implemented in the Hypothesis testing using the Phylogenies (HYPHY; [27]) package. The analyses were performed on web-based Datamonkey interface (http://datamonkey.org/; [28]). The mean d_N_/d_S_ ratio and 95% confidence interval were computed based on Neighbor-Joining (NJ) trees under the HKY85 substitution model [29]. The ratio of the nonsynonymous substitution rate (d_N_) to the synonymous rate (d_S_) was interpreted as follows: d_N_/d_S_ = natural selection; d_N_/d_S_ >1 = positive selection, while d_N_/d_S_ <1 = negative selection. The significance level for selection in both analyses was assessed based on the p-value. P-values <0.05 were considered as thresholds for strong evidence of selection. Phylogenetic relationships were inferred using MrBayes software v3.2 [30] and the generated tree visualized using Fig Tree v1.4.0 software [31].

### Nucleotide Sequence accession numbers

Nucleotide sequences of the partial VP1 genes of the EV68 isolates reported in this study are available in GenBank under accession numbers: KJ472878 to KJ472890.

## Results

Thirteen (13) EV68 isolates were identified. The patients from whom the viruses were detected ranged in age from 2 months to 6 years. The median age was 22 months. The male: female ratio was 10∶3. Majority of the patients (n = 92.3%) were <5 years. Common signs and symptoms the patients exhibited included cough (100%), runny nose (100%), stuffy nose (46%), malaise (46%), vomiting (31%), fatigue (31%) or others including chills, headache and neurological condition (Table 1).

**Table 1:** Demographic and clinical symptoms of the 13 patients in whom EV68 virus was detected.

Patient	Accessionnumber.	Strain name	Age	Sex	Year of samplecollection	Source	DistrictHospital	Clinical details
1	KJ472888	HEV-PDH-016-008	4 y, 1 m	M	2008	NPS	Port Reitz	Cough, Runny and stuffy nose
2	KJ472889	HEV-PDH-038-008	4 y, 4 m	M	2008	NPS	Port Reitz	Cough, Runny and stuffy nose
3	KJ472878	HEV-KCH-044-008	4 y	M	2008	NPS	Kericho	Cough, Malaise, Fatigue, Abdominal pain, Runny and stuffy nose
4	KJ472879	HEV-PDH-082-008	10 m	M	2008	NPS	Port Reitz	Cough, Runny and stuffy nose, Vomiting
5	KJ472880	HEV-PDH-085-008	1, 2 m	F	2008	NPS	Port Reitz	Cough, Runny nose
6	KJ472881	HEV-KSI-112-009	3 y	M	2009	NPS	Kisii	Cough, Chills, Malaise, Fatigue, Runny nose, Headache
7	KJ472890	HEV-KSI-117-010	1 y, 2 m	M	2010	NPS	Kisii	Cough, Malaise, Runny nose, Fatigue
8	KJ472883	HEV-MDH-124-010	1 y, 2 m	F	2010	NPS	Malindi	Cough, Runny and stuffy nose, Vomiting
9	KJ472884	HEV-ALH-126-010	6 y	M	2010	NPS	Alupe	Cough, Chills, Malaise, Neurological, Runny and stuffy nose, Headache, Vomiting, Fatigue
10	KJ472885	HEV-MBG-137-010	2 m	M	2010	NPS	Mbagathi	Cough, Malaise, Runny nose
11	KJ472882	HEV-IDH-156-010	5 m	M	2010	NPS	Isiolo	Cough, Runny nose,
12	KJ472886	HEV-PDH-196-011	1 y 10 m	F	2011	NPS	Port Reitz	Cough, Malaise, Runny and stuffy nose
13	KJ472887	HEV-IDH-199-011	3 y, 3 m	M	2011	NPS	Port Reitz	Cough, Malaise, Neurological, Vomiting, Runny nose,

y = years; m = months; M = male; F = female; NPS = nasopharyngeal swab.

**Figure 1:**
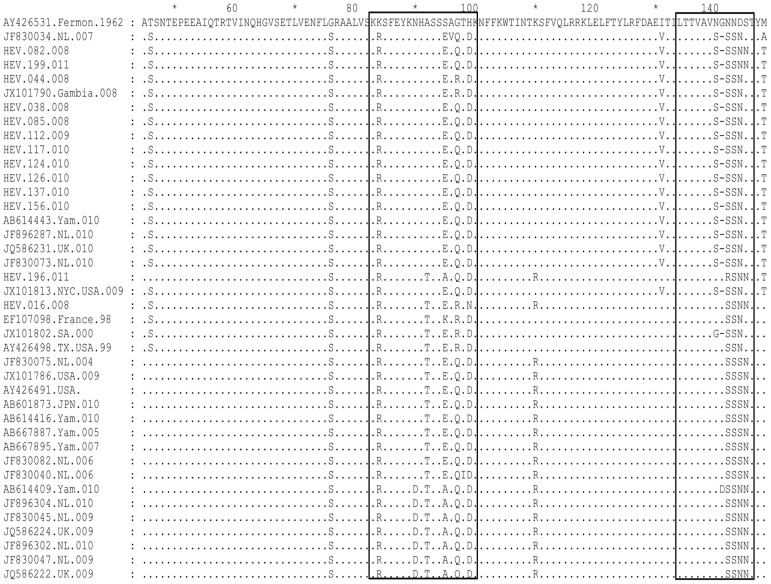
VP1 amino acid sequence alignment of Kenyan EV68 isolates alongside those retrieved from GenBank. Kenyan strains are designated starting with HEV and the other strains bear GenBank accession numbers. The BC and DE-loops are boxed.

**Figure 2:**
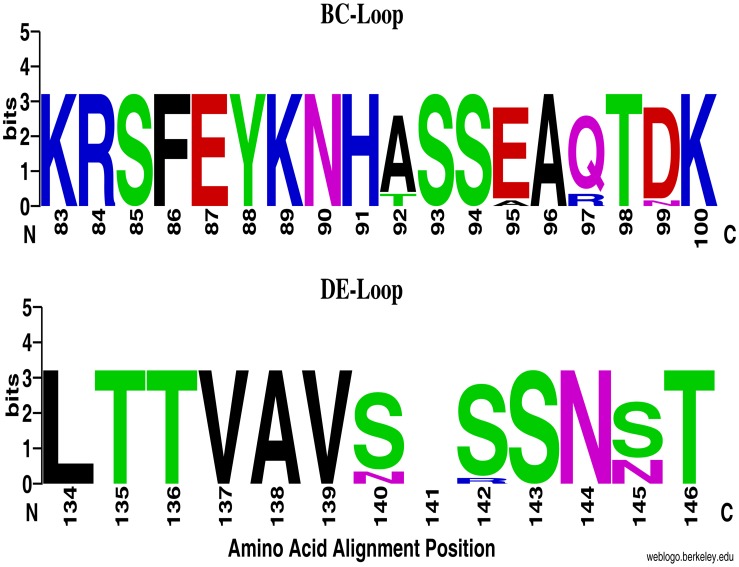
WebLogo comparisons of deduced amino acid sequences within BC and DE-loops of Kenyan EV68 isolates. Each logo is composed of stacks of symbols, one stack for each position in the sequence. The height of the stack indicates the relative frequency of each amino acid at that position. This graphical representation was created using WebLogo available at weblogo.berkeley.edu.

Sequence homology comparison based on the VP1 region showed that Kenyan EV68 strains shared 85.94–87.50% (nucleotide) and 86.79–88.68% (amino acid) sequence identities with the prototype strain (Fermon) (Table 2). Sequence homology among the isolates ranged between 88.12–100% (nucleotides) and 91.51–100% (amino acids). All the Kenyan isolates displayed unique substitutions at four amino acid residue positions (G76S, K84R, N143S and D144N) relative to the prototype Fermon strain (Figure 1). A high proportion (85–92%) of the isolates had further variations at positions T46S, S95E/A, G97Q/R, H99D, I131V, N140S, N142S/R and M148T (Figure 1). Moreover, two Kenyan strains designated HEV-016-008 and HEV-196-011 had an additional A92T amino acid change (Figure 1). Among the amino acid differences, K84R, A92T, S95E/A, G97Q/R and H99D substitutions, were present within the BC loop while N140S, N142S, N143S/R, N144S and S145N were in the DE-loop (Figure 1). Furthermore, the majority (85%) of Kenyan strains had a glycine amino acid deletion at position 141. This occurred as a result of deletion of three nucleotides ATG (Figure 1). All the amino acid substitutions at residue positions G76S, K84R, N142S and N143S in the VP1 region of Kenyan isolates were conserved in sequences of EV68 strains retrieved from GenBank (Figure 1). Likewise, protein mutations at residue positions, T46S, A92T, S97R/Q, K110R, G141, D144N, S145N and M148T were present in the majority of the non-Kenyan strains (Figure 1). Figure 2 depicts consensus amino acid sequences within the BC and DE-loops of Kenyan EV68 strains.

**Table 2:** Intra-isolate sequence homology of the VP1 genomic region of Kenyan EV68 isolates.

	Fermon	HEV-016-008	HEV-044-008	HEV-082-008	HEV-038-010	HEV-085-008	HEV-112-009	HEV-117-010	HEV-124-010	HEV-126-010	HEV-137-010	HEV-156-010	HEV-196-011	HEV-199-011
Fermon		87.5	86.25	86.56	86.25	86.56	85.94	86.88	86.56	86.56	86.25	86.56	85.94	86.88
HEV-016-008	88.68		89.69	89.69	88.12	88.12	89.06	88.12	88.12	88.12	88.44	88.12	94.69	89.38
HEV-044-008	87.74	92.45		97.48	97.16	97.79	97.48	97.79	97.79	97.79	97.48	97.16	89.06	97.16
HEV-082-008	86.79	92.45	98.1		98.42	98.42	98.74	98.42	98.42	98.42	98.11	98.42	89.06	99.68
HEV-038-010	87.74	91.51	99.05	99.05		98.11	98.42	98.11	98.11	98.11	97.79	97.48	87.5	98.11
HEV-085-008	87.74	91.51	99.05	99.05	100		98.42	98.74	100	100	99.68	99.37	88.12	98.11
HEV-112-009	87.74	91.51	99.05	99.05	100	100		98.42	98.42	98.42	98.11	98.11	89.06	98.42
HEV-117-010	87.74	91.51	99.05	99.05	100	100	100		98.74	98.74	98.42	98.11	88.12	98.11
HEV-124-010	87.74	91.51	99.05	99.05	100	100	100	100		100	99.68	99.37	88.12	98.11
HEV-126-010	87.74	91.51	99.05	99.05	100	100	100	100	100		99.68	99.37	88.12	98.11
HEV-137-010	87.74	91.51	99.05	99.05	100	100	100	100	100	100		99.05	88.44	97.79
HEV-156-010	87.74	91.51	99.05	99.05	100	100	100	100	100	100	100		88.44	98.11
HEV-196-011	88.68	94.34	90.57	92.45	91.51	91.51	91.51	91.51	91.51	91.51	91.51	91.51		88.75
HEV-199-011	86.79	92.45	98.1	100	99.05	99.05	99.05	99.05	99.05	99.05	99.05	99.05	92.45	

Numbers at the upper and lower side of the diagonal line indicate homology of nucleotides and amino acids, respectively.

Global selective pressure on the determinant encoding Kenyan EV68 VP1 region was estimated to be 0.0925. This indicated that the bulk of amino acid residues in this part of the viral genomic were under purifying (negative) selection. Indeed, no positively selected sites were detected by either the SLAC or FEL methods. Instead, both methods identified varied number of residues as negatively selected. SLAC method detected 2 amino acid residues (codon positions 86 and 137 relative to Fermon) as evolving under negative pressure with strong statistical significance (p-value <0.05), whereas FEL detected 4 residues under negative pressure at codons 59, 86, 137 and 139 with a p-value <0.05 (Table 3).

**Table 3:** Negative selection pressure analyses at specific codons of the VP1 region of Kenyan EV68 isolates.

		Residue changes					
		From		To			
Analysis Method	Residue positions	Codon	Amino acid	Codon	Amino acid	Normalized dN-dS	P-values
SLAC	**86**	**TTC**	**Phe**	**TTT**	**Phe**	**−27.534**	**0.025**
		**GTG**	**Val**	**GTA**	**Val**		
	**137**	**GTG**	**Val**	**GTC**	**Val**	**−24.285**	**0.037**
		**GTG**	**Val**	**GTA**	**Val**		
FEL	43	GGC	Gly	GGT	Gly	**−**11.706	0.085
	45	GCA	Ala	GCG	Ala	**−**18.027	0.05
	**59**	**ACA**	**Thr**	**ACG**	**Thr**	**−25.012**	**0.034**
		**ACG**	**Thr**	**ACA**	**Thr**		
	69	ACA	Thr	ACT	Thr	**−**20.265	0.069
	76	CTT	Leu	CTC	Leu	**−**14.185	0.097
	79	TTC	Phe	TTT	Phe	**−**12.569	0.088
	81	GTA	Val	GTG	Val	**−**14.939	0.08
		**TTT**	**Phe**	**TTC**	**Phe**		
	**86**	**TTT**	**Phe**	**TTC**	**Phe**	**−101.838**	**0.001**
		**TTC**	**Phe**	**TTT**	**Phe**		
	113	GTG	Val	GTA	Val	**−**16.685	0.066
	119	CTG	Leu	TTA	Leu	**−**20.617	0.057
		CTG	Leu	CTA	Leu		
	122	TTC	Phe	TTT	Phe	**−**17.463	0.085
		**GTG**	**Val**	**GTA**	**Val**		
	**137**	**GTG**	**Val**	**GTC**	**Val**	**−24.413**	**0.002**
		**GTG**	**Val**	**GTA**	**Val**		
	**139**	**GTA**	**Val**	**GTG**	**Val**	**−24.413**	**0.019**
		**GTA**	**Val**	**GTG**	**Val**		
	143	AGT	Ser	AGC	Ser	**−**11.884	0.094
	145	AGT	Ser	AGC	Ser	**−**123.695	0.076
	149	GGC	Gly	GGT	Gly	**−**9.9215	0.096

Codons that showed significant negative selection pressure are indicated in bold type.

**Figure 3:**
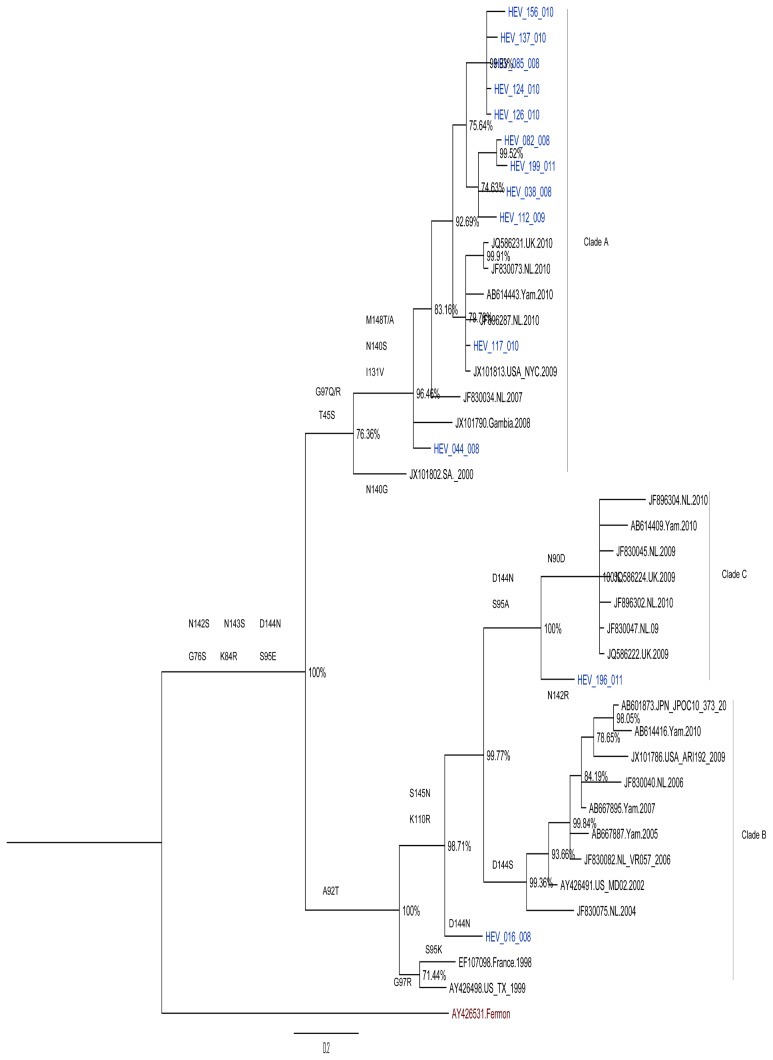
Bayesian phylogenetic tree based on nucleotide sequences of partial VP1 genomic region of EV68 strains. Each reference strain sequence used in the analysis is represented by its GenBank accession number. The tree was estimated using MrBayes 3.2 with a general time-reversible (GTR) substitution model. Posterior probabilities support values are shown as percentages on each node. The scale bar indicates number of nucleotide substitutions per site. Kenyan isolates are shown in blue.

Phylogenetic analysis of the Kenyan isolates with EV68 strains retrieved from GenBank based on the VP1 region revealed separation of the virus isolates into three main clusters A, B and C (Figure 3). Majority (84.6%) of the Kenyan strains belonged to clade A while a minority belonged to clades B and C. The Kenyan strains belonging to clade A clustered closely with sequences of strains from countries such as Gambia (2008), Senegal (2010), the United States (2009), the United Kingdom (2010), New Zealand (2010), Netherlands (2007, 2010), Japan (2010), China (2010), Italy (2008), South Africa (2000), and Thailand (2011). Two of the Kenyan EV68 strains designated HEV-016-008 and HEV-196-011 belonging to clades B and C respectively, were segregated from reference sequences in these groups due to A92T amino acid change. All Kenyan isolates as well as the other global strains used in the analysis had evolutionarily diverged from EV68 Fermon prototype strain.

## Discussion

Since 2008, increasing episodes of EV68 infection have been reported worldwide [3], [5], [8], [10]. This has partly been attributed to improved surveillance systems in some quarters, but data from other work have suggested the virus may have re-emerged [2], [10]. Keeping with these observations, here we report detection of 13 EV68 strains among human enteroviruses isolated in Kenya between 2008 and 2011. Majority of the patients who tested positive for EV68 presented with symptoms associated with mild respiratory illnesses such as cough, runny nose, chills, headache, fatigue among others. Whereas symptoms were recorded for all the patients from whom the viruses were isolated, clinical diagnoses were not provided by the clinicians, hence it was not possible for us to relate amino acid changes in the carboxyl end of VP1 protein of the isolates and virulence (severity of illness). However, all the patients exhibited signs and symptoms associated with mild respiratory illnesses, indicating that EV68 virus is an important etiological agent of ILI respiratory disease in children in Kenya. Two patients presented with neurological symptoms. This was not surprising since EV68 have in rare cases been associated with infections of the central nervous system [3], [12].

Sequence homology analyses revealed sixteen amino acid substitutions in the VP1 region of the Kenyan EV68 strains relative to the prototype strain (Fermon). 25% of these mutations were within the BC-loop of all the Kenyan strains while the majority contained 31% of the changes in the DE-loop. This result echoed findings of similar studies reported in Italy and the Netherlands [10], [32]. The BC and DE-loops are important immunogenic regions associated with enteroviral infectious properties [10]. They are found on the virion surface and are believed to harbor antigenic properties [18], [33]. Amino acid residue substitutions in these loops, especially those resulting in conformational changes, can significantly alter the host’s neutralizing reactivity to the virus [14], [18], [33]–[36]. Thus, the substitutions in the VP1 protein amongst the Kenyan EV68 viruses indicate that they are antigenic variants of the prototype. Consequently, had a vaccine formulation for EV68 based on the prototype strain targeting these epitopes been used in Kenya during the study period, such a vaccine would probably have been ineffective.

A high proportion of protein mutations observed in the VP1 region of Kenyan isolates were also present in contemporaneous global EV68 strains. The presence of common mutations in the VP1 region of both Kenyan and global strains suggests a worldwide transmissibility of these viruses. It also supports the linkage of sequence variation within the VP1 gene of EV68, to increased detection of these viruses [10], [37]. Moreover, the presence of shared mutations within the BC-loop suggests the VP1 region may be a potential target for future vaccine development.

Phylogenetic analysis revealed that majority of Kenyan EV-68 strains belonged to clade A while a minority belonged to clades B and C (Figure 3). This result corroborates findings by Tokarz *et al.,* (2012) [4] who showed worldwide circulation of three primary clades (A, B, C) of EV68. Consistent with previous findings, the Kenyan strains belonging to clade A were characterized by a glycine deletion at residue position 141 [4], [10], [13]. Those belonging to clades B and C were segregated from other strains in their clusters, suggesting that they were distinct sub-lineages. All the Kenyan EV-68 strains as well as those circulating elsewhere during this period had genetically evolved from the prototype strain.

Natural selection analyses indicated that the mean dN/dS (ω) value of the Kenyan viruses were under negative purifying selection in the VP1 region. Indeed, no positively selected sites were detected in this region, through SLAC and FEL analyses. However, disparate numbers of negatively selected sites were identified with strong statistical supports (p- value <0.05). FEL detected more sites under negative selection compared to SLAC. This was not surprising since the FEL method is more powerful than SLAC, because SLAC is conservative [38]. This result echoes findings by Linsuwanon *et al.*, (2012) [14] and suggests that protein mutations observed in the VP1 region of Kenyan isolates were driven by point mutations [39].

This work had a few limitations. First, the lack of clinical diagnoses for patients from whom the viruses were isolated hampered any attempts to associate mutations observed in individual virus isolates with diseases severity to indicate any relationship between strain virulence and mutations in the BC and DE-loops of the VP1 protein. Secondly, since it was a retrospective study relying on isolates obtained previously, it is possible that some EV68 strains may have failed to grow in culture hence leading to an underestimation of the prevalence of these viruses. Finally, the sole use of the VP1 gene to analyze the viruses was limiting since it obscures the possibility of recombination events. Reports of recombination events among human enteroviruses are well established [40]. Despite these shortcomings, we have demonstrated that EV68 strains isolated in Kenya were highly similar to those circulating in other countries, but genetically and antigenically divergent from the prototype strain (Fermon). Findings from this study have also indicated that evolution in the VP1 gene may be contributing to increasing worldwide detection of EV68. However, further genome studies are required, to provide more insight into the genetic and evolutionary characteristics of the Kenyan isolates.
